# Isopropyl 3,4-dihy­droxy­benzoate

**DOI:** 10.1107/S1600536811044965

**Published:** 2011-11-09

**Authors:** Xu-Ji Shen, Qun-Zheng Zhang, Shi-Xiang Wang, Ya-Jun Zhang, Xiao-Hui Zheng

**Affiliations:** a College of Life Sciences, Northwest University, Xi’an 710069, People’s Republic of China; b College of Chemistry & Chemical Engineering, Xian Shiyou University, Xi’an 710065, People’s Republic of China; c College of Life Sciences and Key Laboratory of Resource Biology and Biotechnology in Western China, Ministry of Education, Northwest University, Xi’an 710069, People’s Republic of China

## Abstract

In the crystal structure of the title compound, C_10_H_12_O_4_, O—H⋯O hydrogen bonds incorporating *R*_2_^2^(10) and *R*_2_^2^(14) motifs link mol­ecules into chains along [1
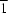
0]. An intra­molecular O—H⋯O hydrogen bond is also observed.

## Related literature

The title compound is a derivative of protocatechuic acid (3,4-dihy­droxy­benzoic acid). For the properties of esters of protocatechuic acid, see: Shizuka *et al.* (2004 ▶); Yun-Choi *et al.* (1996 ▶); Robert *et al.* (1986 ▶). For hydrogen-bond motifs, see: Bernstein *et al.* (1995 ▶).
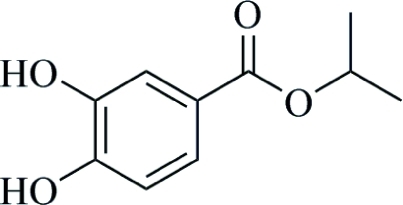


## Experimental

### 

#### Crystal data

C_10_H_12_O_4_
*M* *_r_* = 196.20
Triclinic, 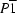

*a* = 5.8485 (12) Å
*b* = 9.1844 (17) Å
*c* = 9.9834 (19) Å
α = 72.629 (3)°
β = 80.547 (3)°
γ = 78.980 (3)°
*V* = 499.06 (17) Å^3^
*Z* = 2
Mo *K*α radiation
μ = 0.10 mm^−1^
*T* = 296 K
0.37 × 0.25 × 0.15 mm

#### Data collection

Bruker APEXII CCD diffractometer
2520 measured reflections
1745 independent reflections
1289 reflections with *I* > 2σ(*I*)
*R*_int_ = 0.012

#### Refinement

*R*[*F* ^2^ > 2σ(*F* ^2^)] = 0.048
*wR*(*F* ^2^) = 0.133
*S* = 1.04
1745 reflections
131 parameters
H-atom parameters constrained
Δρ_max_ = 0.13 e Å^−3^
Δρ_min_ = −0.23 e Å^−3^

### 

Data collection: *APEX2* (Bruker, 2009 ▶); cell refinement: *SAINT* (Bruker, 2009 ▶); data reduction: *SAINT*; program(s) used to solve structure: *SHELXS97* (Sheldrick, 2008 ▶); program(s) used to refine structure: *SHELXL97* (Sheldrick, 2008 ▶); molecular graphics: *SHELXTL* (Sheldrick, 2008 ▶) and *PLATON* (Spek, 2009 ▶); software used to prepare material for publication: *SHELXTL*.

## Supplementary Material

Crystal structure: contains datablock(s) I, global. DOI: 10.1107/S1600536811044965/lh5339sup1.cif

Structure factors: contains datablock(s) I. DOI: 10.1107/S1600536811044965/lh5339Isup2.hkl

Supplementary material file. DOI: 10.1107/S1600536811044965/lh5339Isup3.cml

Additional supplementary materials:  crystallographic information; 3D view; checkCIF report

## Figures and Tables

**Table 1:** Hydrogen-bond geometry (Å, °)

*D*—H⋯*A*	*D*—H	H⋯*A*	*D*⋯*A*	*D*—H⋯*A*
O3—H3⋯O4^i^	0.82	2.15	2.844 (2)	142
O3—H3⋯O4	0.82	2.28	2.720 (2)	115
O4—H4⋯O2^ii^	0.82	1.93	2.747 (2)	175

Symmetry codes: (i) 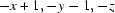; (ii) 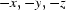.

**Fig. 1.**
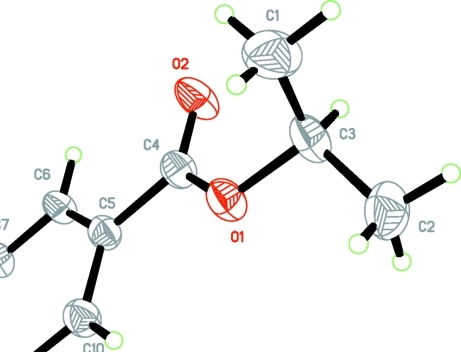
The molecular structure of (I) showing displacement ellipsoids at the 30% probability level.

**Fig. 2.**
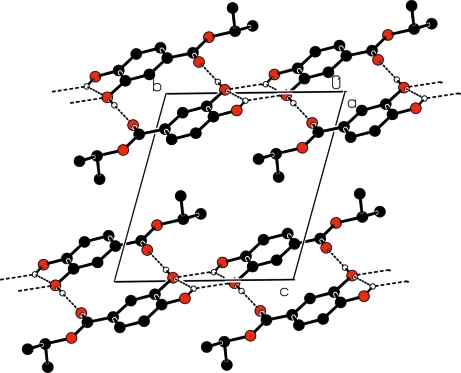
Part of the crystal structure of (I) with Hydrogen bonds shown as dashed lines.

## supplementary crystallographic information

### Comment

Esters of protocatechuic acid  has been shown to have, a DPPH radical scavenging ability, anti-thrombotic activity, and can act as inhibitors of the *sn*-glycerol-3-phosphate oxidase of *Trypanosoma brucei brucei* (Shizuka *et al.*, 2004; Yun-Choi *et al.*, 1996; Robert *et al.*, 1986).

The molecular structure of the title compound (I) is shown in Fig. 1. Intramolecular O—H···O hydrogen bonds form *R*^2^_2_(10) and *R*^2^_2_(14) motifs (Bernstein *et al.*, 1995). In the crystal, intermolecular O—H···O hydrogen bonds link molecules into chains propagating along [110] (see Fig. 2).

### Experimental

To a solution of 0.1*M* protocatechuic acid in 500 ml of 2-propanol at room temperature, 0.01*M* TsOH in 2-propanol was added. After the solution had been allowed to stir and reflux for 16 h, the solvent was removed under reduced pressure. The residue was extracted with ethyl acetate three times and filtered. The filtrate was washed successively with dilute saturated aqueous NaHCO_3_ solution, saturated aqueous NaCl, dried over MgSO_4_, and evaporated. The crude product was purified by chromatography (SiO_2_; elution with petroleum ether-acetoacetate, 6:1 *v*/*v*). Yield 30%. X-ray quality crystals were grown from a solution of the title compound in acetone and toluene at room temperature. Spectroscopic analysis: IR(KBr, χm^-1^): 3458, 3314, 2985, 2957, 1677, 1609, 1531, 1445, 1378, 1347, 1299, 1238, 1165, 1101; ^1^H NMR (DMSO, δ, p.p.m.): 9.539 (s, 1 H), 9.536 (s, 1 H), 7.363—7.366(d, 1 H), 7.299—7.316 (dd, 1 H), 6.804—7.818 (d, 1 H), 5.037—5.079(m, 1 H), 1.285 (s, 3 H), 1.275 (s, 3 H).

### Refinement

All H atoms were visible in difference maps but were included in calculated positions with C—H = 0.93 - 0.98Å, O—H = 0.82 Å, and with *U*_iso_(H) = 1.2*U*_eq_(C) or 1.2*U*_eq_(C_methyl_,O).

### Crystal data

**Table:**

C_10_H_12_O_4_	*Z* = 2
*M**_r_* = 196.20	*F*(000) = 208
Triclinic, *P*1	*D*_x_ = 1.306 Mg m^−^^3^
Hall symbol: -P 1	Melting point: 407(1) K
*a* = 5.8485 (12) Å	Mo *K*α radiation, λ = 0.71073 Å
*b* = 9.1844 (17) Å	Cell parameters from 666 reflections
*c* = 9.9834 (19) Å	θ = 2.4–24.2°
α = 72.629 (3)°	µ = 0.10 mm^−^^1^
β = 80.547 (3)°	*T* = 296 K
γ = 78.980 (3)°	Needle, colorless
*V* = 499.06 (17) Å^3^	0.37 × 0.25 × 0.15 mm

### Data collection

**Table:**

Bruker APEXII CCD diffractometer	1289 reflections with *I* > 2σ(*I*)
Radiation source: fine-focus sealed tube	*R*_int_ = 0.012
graphite	θ_max_ = 25.1°, θ_min_ = 2.2°
φ and ω scans	*h* = −6→6
2520 measured reflections	*k* = −10→10
1745 independent reflections	*l* = −9→11

### Refinement

**Table:**

Refinement on *F*^2^	Primary atom site location: structure-invariant direct methods
Least-squares matrix: full	Secondary atom site location: difference Fourier map
*R*[*F*^2^ > 2σ(*F*^2^)] = 0.048	Hydrogen site location: inferred from neighbouring sites
*wR*(*F*^2^) = 0.133	H-atom parameters constrained
*S* = 1.04	*w* = 1/[σ^2^(*F*_o_^2^) + (0.0685*P*)^2^ + 0.037*P*] where *P* = (*F*_o_^2^ + 2*F*_c_^2^)/3
1745 reflections	(Δ/σ)_max_ < 0.001
131 parameters	Δρ_max_ = 0.13 e Å^−^^3^
0 restraints	Δρ_min_ = −0.23 e Å^−^^3^

### Special details

**Table:**

Geometry. All e.s.d.'s (except the e.s.d. in the dihedral angle between two l.s. planes) are estimated using the full covariance matrix. The cell e.s.d.'s are taken into account individually in the estimation of e.s.d.'s in distances, angles and torsion angles; correlations between e.s.d.'s in cell parameters are only used when they are defined by crystal symmetry. An approximate (isotropic) treatment of cell e.s.d.'s is used for estimating e.s.d.'s involving l.s. planes.
Refinement. Refinement of *F*^2^ against ALL reflections. The weighted *R*-factor *wR* and goodness of fit *S* are based on *F*^2^, conventional *R*-factors *R* are based on *F*, with *F* set to zero for negative *F*^2^. The threshold expression of *F*^2^ > σ(*F*^2^) is used only for calculating *R*-factors(gt) *etc*. and is not relevant to the choice of reflections for refinement. *R*-factors based on *F*^2^ are statistically about twice as large as those based on *F*, and *R*- factors based on ALL data will be even larger.

### Fractional atomic coordinates and isotropic or equivalent isotropic displacement parameters (Å^2^)

**Table:**

	*x*	*y*	*z*	*U*_iso_*/*U*_eq_	
O1	0.2571 (2)	0.15042 (14)	0.29888 (13)	0.0554 (4)	
O2	−0.0069 (2)	0.13603 (15)	0.16625 (15)	0.0666 (5)	
O3	0.7691 (2)	−0.42927 (15)	0.09653 (16)	0.0672 (5)	
H3	0.7088	−0.4623	0.0452	0.101*	
O4	0.3545 (3)	−0.32701 (15)	−0.01531 (15)	0.0667 (5)	
H4	0.2461	−0.2741	−0.0586	0.100*	
C1	−0.0633 (5)	0.2409 (3)	0.4524 (3)	0.0925 (9)	
H1A	−0.1650	0.1839	0.4288	0.139*	
H1B	−0.1541	0.3308	0.4754	0.139*	
H1C	0.0143	0.1771	0.5321	0.139*	
C2	0.2856 (5)	0.3780 (3)	0.3562 (3)	0.0811 (7)	
H2A	0.3599	0.3182	0.4385	0.122*	
H2B	0.2030	0.4736	0.3715	0.122*	
H2C	0.4024	0.3988	0.2762	0.122*	
C3	0.1158 (4)	0.2893 (2)	0.3288 (2)	0.0605 (6)	
H3A	0.0369	0.3511	0.2462	0.073*	
C4	0.1778 (3)	0.0861 (2)	0.21617 (18)	0.0471 (5)	
C5	0.3358 (3)	−0.05140 (19)	0.19011 (18)	0.0436 (4)	
C6	0.2685 (3)	−0.12546 (19)	0.10392 (18)	0.0463 (5)	
H6	0.1255	−0.0895	0.0673	0.056*	
C7	0.4099 (3)	−0.25143 (19)	0.07177 (18)	0.0469 (5)	
C8	0.6225 (3)	−0.3068 (2)	0.12786 (19)	0.0485 (5)	
C9	0.6887 (3)	−0.2345 (2)	0.2151 (2)	0.0553 (5)	
H9	0.8301	−0.2720	0.2534	0.066*	
C10	0.5479 (3)	−0.1072 (2)	0.2463 (2)	0.0521 (5)	
H10	0.5949	−0.0590	0.3047	0.063*	

### Atomic displacement parameters (Å^2^)

**Table:**

	*U*^11^	*U*^22^	*U*^33^	*U*^12^	*U*^13^	*U*^23^
O1	0.0583 (9)	0.0500 (8)	0.0628 (8)	0.0063 (6)	−0.0190 (7)	−0.0256 (6)
O2	0.0620 (9)	0.0635 (9)	0.0807 (10)	0.0197 (7)	−0.0336 (8)	−0.0342 (8)
O3	0.0612 (9)	0.0555 (8)	0.0889 (11)	0.0185 (7)	−0.0253 (8)	−0.0338 (7)
O4	0.0740 (11)	0.0547 (8)	0.0803 (10)	0.0211 (7)	−0.0381 (8)	−0.0354 (8)
C1	0.0704 (16)	0.107 (2)	0.116 (2)	−0.0091 (14)	0.0063 (15)	−0.0656 (18)
C2	0.0992 (19)	0.0651 (14)	0.0901 (17)	−0.0163 (13)	−0.0072 (14)	−0.0374 (13)
C3	0.0705 (14)	0.0491 (11)	0.0651 (13)	0.0111 (10)	−0.0207 (11)	−0.0265 (10)
C4	0.0507 (11)	0.0447 (10)	0.0442 (10)	−0.0008 (8)	−0.0102 (8)	−0.0110 (8)
C5	0.0439 (10)	0.0397 (9)	0.0434 (10)	−0.0011 (8)	−0.0072 (8)	−0.0077 (8)
C6	0.0433 (10)	0.0438 (10)	0.0492 (10)	0.0045 (8)	−0.0136 (8)	−0.0115 (8)
C7	0.0518 (11)	0.0398 (10)	0.0491 (10)	0.0002 (8)	−0.0119 (8)	−0.0133 (8)
C8	0.0461 (11)	0.0407 (10)	0.0540 (11)	0.0027 (8)	−0.0091 (8)	−0.0101 (8)
C9	0.0452 (11)	0.0516 (11)	0.0684 (13)	0.0057 (9)	−0.0203 (9)	−0.0166 (9)
C10	0.0521 (11)	0.0488 (11)	0.0580 (11)	−0.0020 (9)	−0.0159 (9)	−0.0172 (9)

### Geometric parameters (Å, °)

**Table:**

O1—C4	1.330 (2)	C2—H2B	0.9600
O1—C3	1.464 (2)	C2—H2C	0.9600
O2—C4	1.215 (2)	C3—H3A	0.9800
O3—C8	1.361 (2)	C4—C5	1.479 (2)
O3—H3	0.8200	C5—C6	1.386 (2)
O4—C7	1.372 (2)	C5—C10	1.389 (3)
O4—H4	0.8200	C6—C7	1.377 (2)
C1—C3	1.497 (3)	C6—H6	0.9300
C1—H1A	0.9600	C7—C8	1.391 (3)
C1—H1B	0.9600	C8—C9	1.380 (3)
C1—H1C	0.9600	C9—C10	1.381 (3)
C2—C3	1.502 (3)	C9—H9	0.9300
C2—H2A	0.9600	C10—H10	0.9300
			
C4—O1—C3	118.03 (14)	O2—C4—O1	123.08 (16)
C8—O3—H3	109.5	O2—C4—C5	123.18 (17)
C7—O4—H4	109.5	O1—C4—C5	113.73 (15)
C3—C1—H1A	109.5	C6—C5—C10	119.21 (16)
C3—C1—H1B	109.5	C6—C5—C4	117.83 (16)
H1A—C1—H1B	109.5	C10—C5—C4	122.95 (17)
C3—C1—H1C	109.5	C7—C6—C5	121.01 (16)
H1A—C1—H1C	109.5	C7—C6—H6	119.5
H1B—C1—H1C	109.5	C5—C6—H6	119.5
C3—C2—H2A	109.5	O4—C7—C6	123.48 (16)
C3—C2—H2B	109.5	O4—C7—C8	116.86 (15)
H2A—C2—H2B	109.5	C6—C7—C8	119.66 (16)
C3—C2—H2C	109.5	O3—C8—C9	119.09 (16)
H2A—C2—H2C	109.5	O3—C8—C7	121.41 (16)
H2B—C2—H2C	109.5	C9—C8—C7	119.49 (16)
O1—C3—C1	108.46 (17)	C8—C9—C10	120.90 (17)
O1—C3—C2	105.88 (17)	C8—C9—H9	119.6
C1—C3—C2	113.43 (18)	C10—C9—H9	119.6
O1—C3—H3A	109.7	C9—C10—C5	119.73 (18)
C1—C3—H3A	109.7	C9—C10—H10	120.1
C2—C3—H3A	109.7	C5—C10—H10	120.1
			
C4—O1—C3—C1	85.7 (2)	C5—C6—C7—C8	0.8 (3)
C4—O1—C3—C2	−152.21 (17)	O4—C7—C8—O3	0.6 (3)
C3—O1—C4—O2	−0.9 (3)	C6—C7—C8—O3	−178.80 (16)
C3—O1—C4—C5	178.76 (15)	O4—C7—C8—C9	179.39 (17)
O2—C4—C5—C6	0.3 (3)	C6—C7—C8—C9	0.0 (3)
O1—C4—C5—C6	−179.44 (15)	O3—C8—C9—C10	178.19 (17)
O2—C4—C5—C10	179.34 (18)	C7—C8—C9—C10	−0.6 (3)
O1—C4—C5—C10	−0.4 (3)	C8—C9—C10—C5	0.5 (3)
C10—C5—C6—C7	−1.0 (3)	C6—C5—C10—C9	0.3 (3)
C4—C5—C6—C7	178.12 (15)	C4—C5—C10—C9	−178.72 (17)
C5—C6—C7—O4	−178.53 (17)		

### Hydrogen-bond geometry (Å, °)

**Table:**

*D*—H···*A*	*D*—H	H···*A*	*D*···*A*	*D*—H···*A*
O3—H3···O4^i^	0.82	2.15	2.844 (2)	142.
O3—H3···O4	0.82	2.28	2.720 (2)	115.
O4—H4···O2^ii^	0.82	1.93	2.747 (2)	175.

Symmetry codes: (i) −*x*+1, −*y*−1, −*z*;  (ii) −*x*, −*y*, −*z*.
